# Analysis of genomic and immune intratumor heterogeneity in linitis plastica via multiregional exome and T‐cell receptor sequencing

**DOI:** 10.1002/1878-0261.13381

**Published:** 2023-03-07

**Authors:** Jin Huang, Guofeng Zhao, Qiu Peng, Xin Yi, Liyan Ji, Jing Li, Pansong Li, Yanfang Guan, Jie Ge, Ling Chen, Runzhe Chen, Xin Hu, Won‐Chul Lee, Alexandre Reuben, P. Andrew Futreal, Xuefeng Xia, Jian Ma, Jianjun Zhang, Zihua Chen

**Affiliations:** ^1^ The Hunan Provincial Key Lab of Precision Diagnosis and Treatment for Gastrointestinal Tumor Xiangya Hospital, Central South University Changsha Hunan China; ^2^ Department of Oncology, Xiangya HospitalXiangya Hospital Central South University Changsha China; ^3^ Department of General Surgery, Xiangya Hospital Central South University Changsha China; ^4^ International Joint Research Center of Minimally Invasive Endoscopic Technology Equipment & Standardization Changsha China; ^5^ National Clinical Research Center for Geriatric Disorders Xiangya Hospital Changsha China; ^6^ Geneplus‐Beijing Institute Beijing China; ^7^ Geneplus‐Beijing Beijing China; ^8^ Cancer Research Institute, School of Basic Medical Science Central South University Changsha China; ^9^ Department of Thoracic and Head and Neck Medical Oncology The University of Texas MD Anderson Cancer Center Houston TX USA; ^10^ Department of Genomic Medicine The University of Texas MD Anderson Cancer Center Houston TX USA

**Keywords:** gastric linitis plastica, intratumor heterogeneity, multiregional whole‐exome sequencing, T‐cell receptor repertoires

## Abstract

The molecular landscape and the intratumor heterogeneity (ITH) architecture of gastric linitis plastica (LP) are poorly understood. We performed whole‐exome sequencing (WES) and T‐cell receptor (TCR) sequencing on 40 tumor regions from four LP patients. The landscape and ITH at the genomic and immunological levels in LP tumors were compared with multiple cancers that have previously been reported. The lymphocyte infiltration was further assessed by immunohistochemistry (IHC) in LP tumors. In total, we identified 6339 non‐silent mutations from multi‐samples, with a median tumor mutation burden (TMB) of 3.30 mutations per Mb, comparable to gastric adenocarcinoma from the Cancer Genome Atlas (TCGA) cohort (*P* = 0.53). An extremely high level of genomic ITH was observed, with only 12.42%, 5.37%, 5.35%, and 30.67% of mutations detectable across 10 regions within the same tumors of each patient, respectively. TCR sequencing revealed that TCR clonality was substantially lower in LP than in multi‐cancers. IHC using antibodies against CD4, CD8, and PD‐L1 demonstrated scant T‐cell infiltration in the four LP tumors. Furthermore, profound TCR ITH was observed in all LP tumors, with no T‐cell clones shared across tumor regions in any of the patients, while over 94% of T‐cell clones were restricted to individual tumor regions. The Morisita overlap index (MOI) ranged from 0.21 to 0.66 among multi‐regions within the same tumors, significantly lower than that of lung cancer (*P* = 0.002). Our results show that LP harbored extremely high genomic and TCR ITH and suppressed T‐cell infiltration, suggesting a potential contribution to the frequent recurrence and poor therapeutic response of this adenocarcinoma.

## Graphical abstract

Integrated by multi‐regional whole‐exome sequencing and T‐cell repertoire sequencing (TCR), we assessed the intratumoral heterogeneity (ITH) and immune status in gastric linitis‐plastica (LP). LP presented extreme ITH at genomic and TCR levels. Immunohistochemistry against CD4, CD8, and PD‐L1 further demonstrated its immune‐cold microenvironment. Altogether, high ITH are potential contributors to the frequent recurrence and poor therapeutic response of LP.

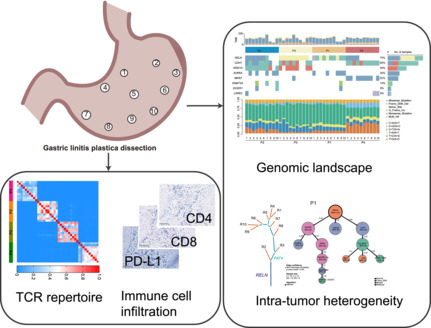

**Abbreviations**
BRCA: breast invasive carcinoma
CRC: colorectal cancer
DFS: disease‐free survival
GC: gastric carcinoma
H&E: hematoxylin and eosin
ITH: intratumor heterogeneity
LP: linitis plastica
LUAD: lung adenocarcinoma
LUSC: lung squamous cell carcinoma
MOI: Morisita overlap index
OS: overall survival
STAD: stomach adenocarcinoma
TCGA: The Cancer Genome Atlas
TCR: T‐cell receptor
TMB: tumor mutation burden
WES: whole‐exome sequencing

## 1 Introduction

Linitis plastica (LP) is a rare and aggressive gastric carcinoma (GC) with unique clinical and pathological features [1, 2]. Distinct from other GC, LP does not grow as a gastric mass but diffusely infiltrates the entire stomach in a desmoplastic pattern [3]. The diffused LP leads to microscopic thickening of the stomach wall and prominent submucosal hypertrophy of the whole stomach. LP is characterized by a high rate of local spread to lymph nodes and peritoneal cavities. Less than 30% of patients may be eligible for surgical resection, and the carcinoma recurs up to 90% of patients post‐surgery [4]. The 5‐year overall survival (OS) is only 4–15% for patients with advanced LP [5]. There is an urgent need to understand the underlying biology and molecular mechanisms associated with the aggressive features and poor clinical outcomes of LP, to guide the development of novel therapeutic strategies.

Intratumor heterogeneity (ITH) contributes to tumorigenesis, aggressiveness and prognosis. ITH may have a profound impact through tumor sampling bias on defining targetable mutations [6]. Multiregional sequencing provides a more accurate methodology for the assessment of genomic heterogeneity than single‐region sequencing does. Genomic ITH has been reported in many cancer types at mutational and copy number variation levels [7, 8, 9, 10] and complex ITH could predict poorer clinical outcomes. Aside from cancer cells, ITH is extended to tumor‐associated cells, including tumor‐infiltrated immune cells. For instance, Alexandre et al. [11] reported that only 5.7% of tumor‐infiltrated T‐cells were present across different regions within the same lung cancer tissues by multiregional T‐cell receptor (TCR) sequencing. In addition, higher ITH was associated with higher risks of postsurgical recurrence and shorter disease‐free survival (DFS) in localized non‐small cell lung cancers (NSCLC) [11]. Due to the scarcity of LP tissues, particularly resected LP tumors, the genomic and immune ITH architectures of LP are unknown.

To understand the landscape and the ITH of LP at genomic and immunologic levels, we performed multiregional whole‐exome sequencing (WES) and TCR sequencing on 40 tumor regions from four patients with resected LP tumors (10 spatially separated regions per tumor). Our results demonstrated extensive genomic and TCR ITH in all four LP tumors.

## 2 Materials and methods

### 2.1 Patients and samples

**Fig. 1:**
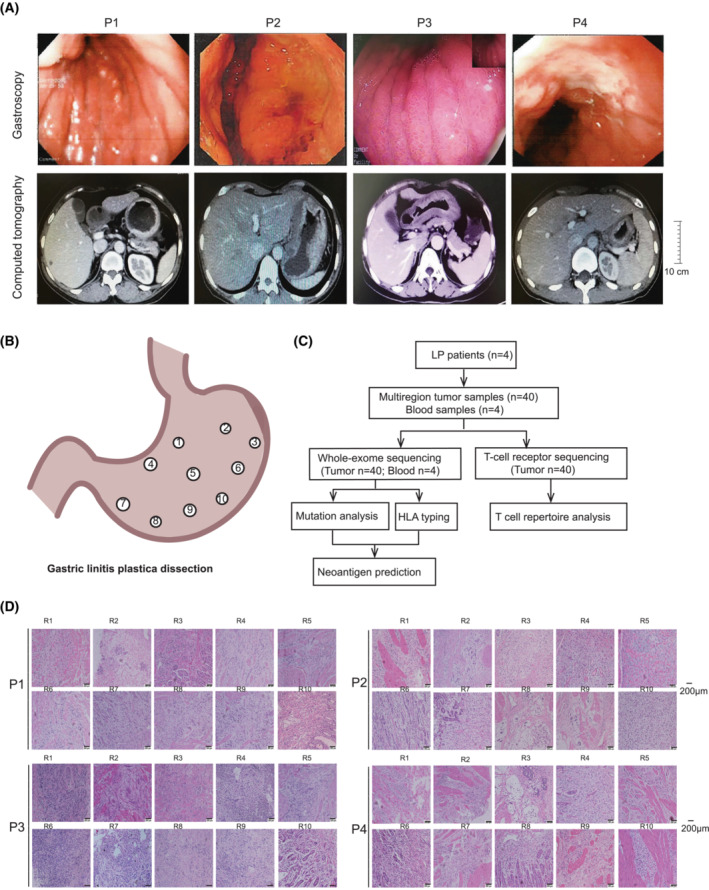
Characterization of gastric linitis plastica and study scheme for multiregional sequencing. (A) Representative endoscopic and computed tomography (CT) images of four LP patients show extensive thickening of the gastric wall. The endoscopy revealed a diffuse infiltrative lesion across the whole corpus and antrum in all 4 LP patients (upper panel). The inset image represents a fully reduced image of diffuse infiltrative lesion across the whole corpus and antrum in P3. CT of all four patients also showed wall thickening of the antrum and corpus of the stomach. Scale bars: 10 cm. (B) Spatial distribution of multiregional specimens obtained from a resected LP tumor (P1) as an example. (C) Representative H&E images of 40 regional specimens from four LP patients. Scale bars: 200 μm. The images show a large number of cancer cells in each tumor region with numerous fibroblasts and cellulose staining in the background. LP, Linitis plastica. (D) Workflow for multiregional whole exome and TCR sequencing.

Multiregional tumor samples and paired peripheral blood were obtained from four patients with LP who underwent radical gastrectomy at the Xiangya Hospital of Central South University from September 2014 to April 2016. Informed consent was obtained from all patients. The study was approved by the Ethics Committee of the Xiangya Hospital of Central South University (IRB# 201312487) and followed the Declaration of Helsinki. The experiments were undertaken with the understanding and written consent of each subject. Representative images of gastroscopy and computed tomography (CT) are shown in Fig. 1A. The four patients were treatment‐naïve before the surgery. Postoperative clinicopathological features (gender, Lauren type, WHO type, TNM stage), biomarker features (HP‐negative, HER2‐negative, EBV‐negative), adjuvant chemotherapy and survival time were listed in Table S1. Immediately after resection, 10 regions from each tumor were collected (*n* = 40) from antrum, body, fundus, pylorus, and major and minor curvatures (Fig. 1B). All tumor specimens were split into two halves with half fresh frozen (for DNA extraction, whole exome sequencing, and TCR sequencing) and the other half fixed in formalin, embedded in paraffin [FFPE specimens for hematoxylin and eosin (H&E) staining and immunohistochemical **(**IHC) staining]. Two experienced cancer pathologists reviewed H&E slides from each sample to assess and confirm the diagnosis and assess the tissue quality. The representative H&E images are shown in Fig. 1C.

### 2.2 DNA extraction

Genomic DNA (gDNA) from tumor tissues and peripheral blood leukocytes (PBL) were isolated using the DNeasy Blood & Tissue Kit (Qiagen, Hilden, Germany) following the manufacturer's instructions. Paired blood DNA samples from the same patients were used as germline control for sequencing.

### 2.3 Whole exome sequencing

Before library construction, 1.0 μg each of gDNA from fresh frozen tissues or blood was sheared into 300‐bp fragments with a Covaris S2 ultrasonicator. Indexed libraries were prepared using the KAPA Library Preparation Kit (Qiagen, Wilmington, MA, USA). According to the manufacturer's instructions, DNA libraries were hybridized to SureSelect Human All Exome v5 reagents (Agilent, Santa Clara, CA, USA). Sequencing was carried out using Illumina 2 × 150 bp paired‐end reads on an Illumina HiSeq X‐Ten instrument according to the manufacturer's recommendations using a TruSeq PE Cluster Generation Kit v3 and a TruSeq SBS Kit v3 (Illumina, San Diego, CA, USA).

### 2.4 Germline variant detection

Low‐quality reads from the original sequencing results were filtered with ncfilter (version: 1.5.0) in‐house software, and filtered reads were aligned to human genome (hg19) with bwa (version: 0.7.15‐R1140; http://github.com/lh3/bwa), and sentieon software was employed to mark repeated reads, modify indel region re‐alignment and quality value. Finally, DNAscope module of sentieon (version: 201808, DFETECH Sentieon Inc., Mountain View, CA, USA) was employed to generate VCF file and then use in‐house ncanno software to annotate pathogenic or likely pathogenic (P/LP) germline mutations according to 2015 ACMG/AMP guidelines [12].

### 2.5 Somatic variant detection

The adaptor sequences and low‐quality reads were removed from the raw data. bwa (version: 0.7.12‐r1039) was employed to align the clean reads to the reference human genome (hg19). picard‐gatk (version: 1.98) was used to mark PCR duplicates. Realignment and recalibration were performed using gatk (version: 3.4‐46‐gbc02625; http://gatk.broadinstitute.org/hc/en-us). Somatic mutations were called as reported previously. [13]

### 2.6 Somatic copy number variation detection

Copy number variation was detected by FACETS algorithm [14]. FACETS determined the allele specific copy number variation, as well as clonal heterogeneity, tumor ploidy and purity by non‐parametric joint segmentation. Segments with median log2 ratio > 1.25 were annotated as copy number gain. Copy number loss was identified as a log2 ratio < 0.75.

### 2.7 Mutational signature analyzing

The deconstructsigs (version: 0.20.6) [15] package was used to extract mutational signature based on a negative matrix factorization (NMF) algorithm, and deconstruct mutational progress in each tumor from LP and TCGA stomach adenocarcinoma. The matlab sigprofiler (version: 1.0.0.0) package [16], which is based on a NMF algorithm, was applied to extract the whole mutational signature through the stability and reconstruction error judgment in each LP patient. The curated mutational signature sets were based on combined COSMICv2 signatures.

### 2.8 Multiregional tumor tree construction

All somatic mutations were used to construct multiregional tumor trees using binary presence/absence matrices according to the regional distribution of variants within the tumor. The r package phangorn (version: 1.99‐7) was utilized to perform the parsimony ratchet method [17], generating unrooted trees. Branch lengths were determined using the Acctran function.

### 2.9 Driver tree construction of somatic mutation and copy number variations

The significant copy number variation and driver mutations were used to construct a tumor progression model using the tronco package [18]. The significant copy number variations were obtained from GISTIC2. [19] Driver mutations were defined as mutations within oncoKB cancer genes (https://www.oncokb.org/cancerGenes).

### 2.10 Clonality construction

The subclonal architecture of all samples from available LP tumors were constructed by PyClone [20]. The copy number variation of each non‐silent SNV was used as input for PyClone analysis and the CCF was inferred and variants were clustered as previously described [21]. PyClone was run with 20 000 iterations and default parameters. To characterize the clonal composition of a tumor, the CCF 95 quartile was calculated for each patient, and the 95 quartiles was used as the threshold. Mutations in the cluster id with the maximal mean cellular prevalence were considered clonal, otherwise mutations were considered as subclonal.

### 2.11 TCR sequencing

Immuno‐sequencing of the CDR3 regions of human TCRβ chains was performed using the multiplex PCR amplification method. To amplify all possible V(D)J combinations, a multiplex PCR1 assay with 32 V forward and 13 J reverse primers were used. Sequencing libraries were loaded onto the Illumina Hiseq3000 System with 151 bp reads. The CDR3 sequence was defined as the amino acids between the second cysteine of the V region and the conserved phenylalanine of the J region according to the international ImMunoGeneTics database (IMGT) V, D and J gene references. The CDR3 sequences were identified and assigned using the MiXCR package [22]. One million normalized reads were randomly selected for further analysis. An average of 1412 clones were obtained in all the samples. Shannon's entropy was calculated on the clonal abundance of all productive TCR sequences. Dividing Shannon's entropy by the natural logarithm of the number of unique productive TCR sequences, we obtained the normalized Shannon's entropy. Clonality was the reciprocal of normalized Shannon's entropy (clonality = 1/normalized entropy) with values ranging from 0 (most diverse) to 1 (least diverse). The Morisita overlap index (MOI) for determining the similarity between samples ranges from 0 and 1, representing minimal and maximal similarity, respectively. The maximal difference in T‐cell clonality is defined as the intratumor difference between the highest and lowest T‐cell clonality across regions within the same tumors.

### 2.12 Immunohistochemistry (IHC)

All tissue sections were subjected to IHC as previously described [23]. In brief, paraffin‐embedded sections were cut 4 μm thick, then deparaffinized and rehydrated. Antigenic retrieval was processed with sodium citrate. The sections were then incubated in 3% H_2_O_2_ for 10 min, blocked in 1% BSA for 60 min followed by immunostaining using anti‐PD‐L1 mAb (Zsbio, Beijing, China, #ZM‐0381, clone UMAB199, 1 : 100 dilution), anti‐CD4 (Zsbio, #ZM‐0418, clone UMAB64, 1 : 75 dilution) and anti‐CD8 (Zsbio, #ZA‐0508, clone EP334, 1 : 75 dilution). The IHC images were quantified by a number of cells·mm^−2^. The most and least filtrated regions were defined as regions with maximum and minimum values, respectively, as determined by IHC quantification.

### 2.13 Statistical analysis

Wilcoxon test in the r‐package ggpubr (version: 0.4.0) was used to calculate the difference between tumor mutation burden (TMB) of gastric cancer in our cohort and that of TCGA, and then the *t*‐test was used to calculate the correlation of trunk proportion of three random samples of each patient. In the analysis of the mutational signature, the similarity was calculated between the COSMIC and predicted signature by the lsa (version: 0.731) r package. Fisher's exact test was used to calculate the trunk percentage difference between mutation and neoantigen.

## 3 Results

### 3.1 Genomic landscape of LP

**Fig. 2:**
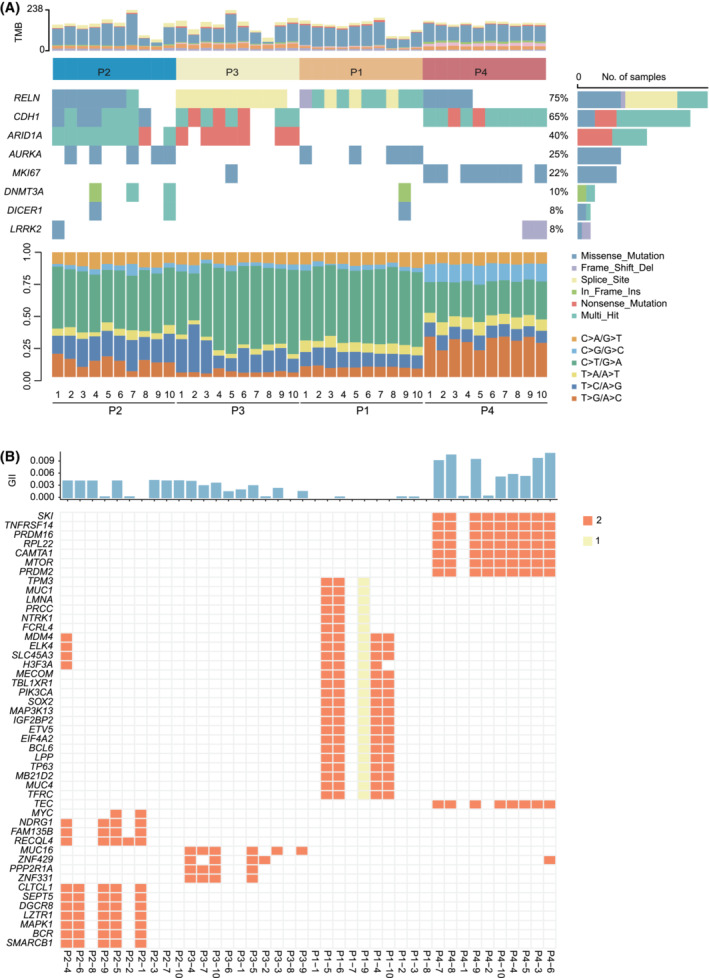
Frequently altered genes in the 40 tumor regions from 4 LP patients. (A) The mutations from the top 30 genes with the highest mutation frequency among the 40 tumor regions are shown with the mutation frequency on the left and the tumor mutation burdens (TMB) at the top (light gray for synonymous mutations and gray non‐synonymous mutations, respectively) and mutation spectrum of transitions (Ti) and transversions (Tv) in 40 tumor regions from four LP tumors on the bottom. Different types of somatic variants are indicated by different colors on the right. (B) Recurrent cancer gene with copy number variation in the four LP patients. The genomic instability index (GII) is denoted by the top barplot column.

In total, 40 spatially distinct tumor regions from four patients with primary LP (Fig. 1D and Table S1) underwent WES at a median sequencing depth of 290.63× (Table S2) and an average purity of 0.3 (Table S3). A total of 11 504 somatic mutations, including 6339 non‐silent mutations, were identified for a median non‐silent TMB of 3.30 mutations per Mb (range: 1.36–4.88 mutations per Mb, Fig. 2), comparable to gastric adenocarcinoma (TCGA) (median: 3.30 vs. 2.84 mutations per Mb, *P* = 0.53; Fig. S1) [10]. The most frequently mutated driver genes were *RELN*, *CDH1* and *ARID1A*, identified in 75%, 65% and 40% of tumor samples, respectively (Fig. 2A). *RELN*, *CDH1* and *ARID1A* mutations were identified in four, three and two LP patients, respectively (Fig. 2A). All tumor regions were microsatellite‐stable as determined by the MSIsensor (Table S1). We did not identify any pathological or likely pathologic germline mutations according to ACMG/AMP guidelines [12].

To assess the fraction of copy number variation in the LP, we calculated the genome instability index for each sample. Copy number variation affected 0.363% (8.33e‐05–1.08%) of the whole genome (Fig. 2B). The recurrent genes with copy number alteration were *ELK4*, *MDM4*, *SLC45A3*, *H3F3A* and *ZNF429*. *MYC* amplification occurred only in patient 4 (Fig. 2B).

### 3.2 Substantial genomic ITH in LP

**Fig. 3:**
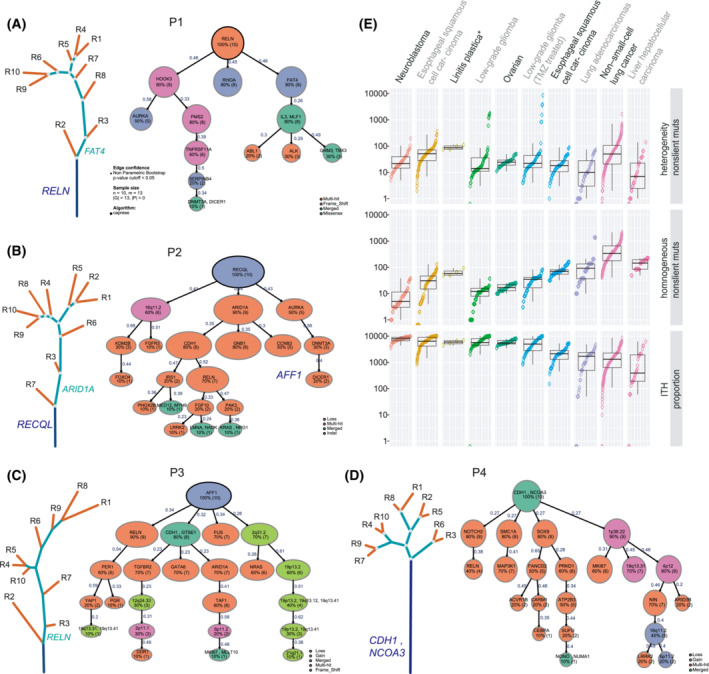
Substantial genomic ITH in LP. (A–D) Phylogenetic trees of four LP patients generated by the clustering of non‐silent somatic mutations identified in multiregional tumor samples. The lengths of trunks/branches were proportional to the number of mutations. Clonal structure using TRONCO package in the four LP tumors. The variant classification was indicated by different colors for each patient. The number of samples carrying the specific gene is shown below the gene for each patient. Blue, green and red lines represent the trunk, branch and private branches, respectively. Candidate driver mutations were mapped to phylogenetic trees. (E) Intratumor heterogeneity of different cancer types depicted by multiregional exome sequencing, measured by standard variation. To minimize the potential confounding effect by a different number of tumor regions per tumor sequenced on ITH, four of the 10 tumor regions of each LP were randomly selected to re‐calculate the proportion of trunk mutations. This analysis was repeated 100 times to compare genomic ITH with different cancer types with two to five tumor regions analyzed.

We next explored the genomic intratumoral ITH of these four LP tumors (Fig. 3A–D). Mutations identified in all regions of each tumor included *RELN* in patient 01, *RECQL* in patient 2, *AFF1* in patient 3, and *CDH1* and *NCOA3* in patient 4. The percentages of mutations identified in all regions within the same tumors were 12.42%, 5.37%, 5.35% and 30.67%. In comparison, 35.85%, 37.42%, 23.55% and 15.33% of mutations were private mutations detectable in only one of the 10 samples within the same tumors (Fig. S2). When comparing the genomic ITH of LP with other cancer types, including neuroblastomas [24], esophageal squamous cell carcinoma [21], low‐grade glioma [25], ovarian cancer [26], low‐grade glioma (TMZ treated) [27], lung cancer [28] and hepatocellular carcinoma [29] depicted by multiregional WES, LP was the most heterogeneous (Fig. 3E).

To further explore fully the clonal structure of LP, we used somatic driver mutations and significant copy number variations to establish evolutionary trajectories by the TRONCO algorithm. Patient 1 harbored *RELN* mutation across all regions, whereas *HOOK3*, *RHOA* and *FAT4* were branch mutations. No significant SCNV event was found in patient 1 (Fig. 3A). In patient 2, *RECQL* indel was a trunk mutation, and branch events included 16q11.2 loss and *ARID1A* mutation (Fig. 3B). *AFF1* frame‐shift was found in all the tumor regions of patient 3. *RELN*, *CDH1*, *FUS* mutations and 2q31.2 gain were branch events in patient 3 (Fig. 3C). Patient 4 had a *CDH1* mutation in the trunk. In contrast, *NOTCH2*, *SMC1A*, *SOX9* and 1p36.22 gain was identified in the branches (Fig. 3D). Although the mutated driver genes were distinct, driver mutations dominantly occurred across all regions in the four LP patients. Conversely, SCNV were identified only in the branches (Fig. 3A–D). These suggested that driver mutations occurred early, whereas SCNV alterations occurred late in the four LP patients.

### 3.3 Distinct mutational processes are observed in different tumor regions

We next derived mutational signatures in each tumor region to depict the ITH of mutagenic processes in LP. Tumor specimens exhibited a predominance of C > T transitions at WpCpG trinucleotides (C > T substitutions in a TCW context, where W = A, G, C or T; Figs 2A and S3). The top mutational signatures in this cohort across different patients included Signature 1 (associated with spontaneous deamination), Signature 6 (associated with defective DNA mismatch repair), Signature 15 (associated with defective DNA mismatch repair), Signature 17 (etiology unknown), etc. (Fig. S4A,B), which were similar to mutational signatures in gastric adenocarcinomas from TCGA [10]. On the other hand, Signature 3 (associated with failure of DNA double‐strand break repair by homologous recombination) was the top mutational signature in LP, primarily driven by patient 4 (Fig. S4), highlighting the critical role of DNA repair defect during mutagenesis gastric cancers. Overall, the dominant signatures in different tumor regions within the same tumors were similar. For each patient, the signatures were shared among different regions within the same tumor, with 68–95% of signatures identifiable across all 10 regions, reflecting the overall similar genetic background and exposure history within each tumor. In patient 3, however, the mutational signatures were highly heterogeneous (Fig. S5A,B), indicating distinct mutational patterns in different tumor regions.

Next, we attempted to understand the mutagenesis during the progression of these 4 LP tumors by analyzing the mutational signatures associated with trunk vs. branch mutations. Due to the tiny number of trunk mutations in each patient, we tried to combine the trunk vs. branch mutations from all four patients for this analysis. With this caveat fully acknowledged, this analysis demonstrated that deamination‐associated Signature 1 remained the top signature in both trunk and branch/private mutations (Fig. S5C), suggesting a persistent role of spontaneous deamination during early and late neoplastic evolution of LP tumors. The DNA double‐strand break‐related Signature 3 was among the top signatures associated with trunk mutations but not branch/private mutations, whereas Signature 6 (DNA mismatch repair) had a substantial contribution to branch/private mutations but not to trunk mutations (Fig. S5D), indicating different mutational processes at play during the chronological progression of the LP tumors.

### 3.4 Substantial immune heterogeneity of LP

**Fig. 4:**
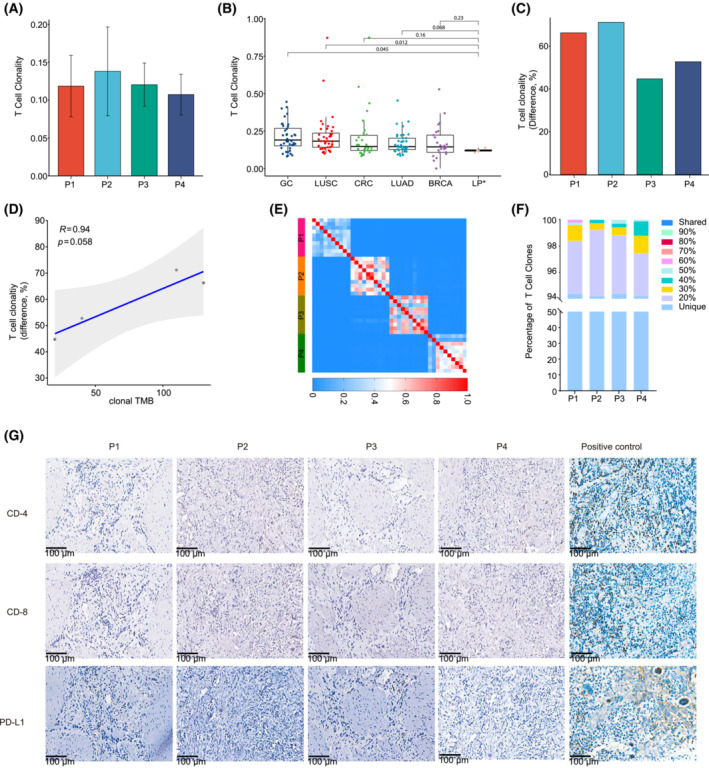
T‐cell repertoire ITH in LP. (A) The barplot showing the T‐cell clonality in each LP tumor. Data are shown as mean ± SD. (B) Comparison of T‐cell clonality in LP with other cancer types. The mean T‐cell clonality from 10 regions of each LP was utilized. The *P*‐value was from the Mann–Whitney *U*‐test. (C) Maximum difference in T‐cell clonality, defined as the intratumor difference between the highest and lowest T‐cell clonality across regions within the same tumors. (D) The Pearson correlation between the maximum difference in T‐cell clonality and clonal TMB of each LP tumor. (E) Quantification of T‐cell receptor ITH by the Morisita index (MOI), a metric taking into consideration not only the composition of T‐cell clones but also the abundance of individual T‐cell clones. MOI ranges from 0 to 1, with 1 indicating identical TCR repertoires and 0 indicating completely distinct TCR repertoires between two samples. The color scales indicate the MOI between two tumor regions. (F) Percentage of T‐cell clones detected in different proportions of tumor regions from the same LP tumors. (G) The IHC staining of CD4, CD8 and PD‐L1 in the four LP tumors. Scale bars: 100 μm. All the regions within each tumor were negative, and representative images and positive control are shown.

In light of our previous work identifying substantial mutational heterogeneity in these LPs, we sought to determine whether the neoantigen landscape mirrors the mutational ITH. We performed an *in silico* prediction of HLA‐A/B/C (MHC I) neoantigens based on mutation data; only 6.16%, 2.17%, 11.98% and 12.42% of predicted neoantigens were associated with trunk mutations, respectively (Fig. S2 **)**. It is known that neoantigen could initiate the tumor‐infiltrated T‐cell response [30]. We next performed multiregional TCR sequencing to assess ITH in the T‐cell response. The average TCR clonality, a metric of T‐cell expansion and reactivity ranging from 0.10 (P4) to 0.14 (P2) (Fig. 4A), was the lowest among six cancer types in the Geneplus in‐house database (Fig. 4B). Even with such a small sample size, the difference was significant between LP and lung squamous cell carcinoma (LUSC) (*P* = 0.012) and gastric carcinoma (GC) (*P* = 0.045). In comparison with the most and least infiltrated regions, TCR clonality varied ranging between 44.74% (P3) and 71.19% (P2) among different tumor regions from the same tumors (Fig. 4C). Interestingly, we found a marginally significant positive correlation between intratumor difference of T‐cell clonality and clonal TMB in LP (*P* = 0.058, *r* = 0.94; Fig. 4D), implying the association of clonal mutational burden and immune microenvironment.

To further quantify the TCR ITH, we utilized the MOI, which considers both the composition and the abundance of T‐cell rearrangements. The average MOI of any pair of tumor regions within each tumor were 0.35 (P1), 0.53 (P2), 0.56 (P3) and 0.44 (P4), respectively (Fig. 4E), significantly lower than those of NSCLC [11] (*P* = 0.002). As expected, none of the T‐cell clones was shared between any two patients. Additionally, none of the T‐cell clones was shared among all tumor regions from the same tumors. Particularly, 94.41%, 94.23%, 94.40% and 94.23% of T‐cell clones were restricted to individual tumor regions from P1, P2, P3 and P4, respectively (Fig. 4F). These results suggested profound T‐cell repertoire heterogeneity.

We then immunostained all the samples using T‐cell markers CD4 and CD8, and the immune checkpoint marker PD‐L1. In line with the TCR repertoire results, scant CD4^+^ (68 cells·mm^−2^) and CD8^+^ (37 cells·mm^−2^) T‐cells within the tumor island or in the stroma and PD‐L1 were negative (< 1%) in all four LP tumors (Fig. 4G), further suggesting the immune‐cold microenvironment with limited and heterogeneous T‐cell infiltration in the four LP tumors.

## 4 Discussion

Linitis plastica is a rare and aggressive cancer with a considerably inferior prognosis. With a poor response to chemotherapy or radiotherapy and an unclear molecular mechanism of therapeutic resistance, complete surgical resection remains the only chance of cure for patients with LP [29]. Translational studies to further broaden our understanding of the cancer biology of LP are urgently needed to provide insight into the development of novel therapeutic strategies. Due to the limited availability of tumor tissues, the molecular landscape and its ITH architecture have not been well studied in LP.

In the current studies, we delineated the genomic and TCR repertoire ITH of four LP tumors using a multiregional profiling approach. Our results demonstrated profound genomic ITH, suggesting that many different cancer‐cell clones with distinct cancer biology are present in these LP tumors. Our results suggest that it is challenging to eradicate completely all cancer cell clones by currently available therapeutic modalities which potentially lead to high risks of recurrence [10].

The frequently mutant *RELN* gene in LP was distinct from those genes (*TP53*, *BRCA2*, *ERBB4*) identified in gastric cancer [31, 32]. *RELN* (Reelin), a key regulator of neuronal migration, is frequently epigenetically silenced in different cancers, including pancreatic [33], breast [34] and gastric cancer [35]. Loss of *RELN* expression was significantly associated with the more advanced stage of GC, and the disruption of the *RELN* pathway might be involved in gastric carcinogenesis [35]. *RAS/PI3‐kinase* controlled *RELN* to promote cell motility and tumor metastases [36]. However, the role of *RELN* in the progression of GC needs to be explored further. *CDH1* and *ARID1A* identified in LP were commonly mutated in gastric cancer [31]. The abnormal expression of *CDH1*, encoding E‐cadherin, is a common early event in gastric tumorigenesis [37]. *CDH1* is frequently mutated in different tumors by genomic sequencing, especially in diffuse‐type tumors along with advanced stages [38]. An integrative analysis showed that *CDH1* was somatically altered in higher proportions in diffuse gastric cancers with younger female patients [39]. *CDH1* mutation was more frequently identified in peritoneal carcinomatosis than in primary gastric adenocarcinoma [40]. Moreover, *CDH1* alteration was significantly associated with shortened survival of GC patients [41]. The germline *CDH1* mutations also confer a high lifetime risk of developing hereditary diffuse gastric cancer (HDGC) syndrome [42, 43]. Taken together, the LP showed a distinct mutational landscape and molecular intratumor heterogeneity with gastric cancer, which might lead to the differential phenotype.

The mechanisms underlying the development of molecular ITH are poorly understood. Cancer progression could be ascribed to the complex interaction between evolving cancer cells (accumulation of somatic mutations, epigenetic aberrations, etc.) and host factors, particularly anti‐tumor immune surveillance. Without effective immune surveillance applying selection pressure, different cancer clones might potentially evolve in parallel as long as there is adequate nutrition and available space, leading to more heterogeneous cancer cell populations [40, 44]. LP is known to be embedded in rich lymphatic vessels and fibroblasts that might potentially provide nutrition to cancer cells [45]. Importantly, these four LP tumors were found to be immune‐cold microenvironments with scant T‐cell infiltration by either TCR sequencing or immunohistochemical staining. Besides, the TCR repertoire in these four LP tumors was extremely heterogeneous. LP tumors are characterized by profound stromal fibrosis around tumor cells during infiltration and proliferation, which is known to serve as a protective shield for cancer cells from attacks by host immune cells in various cancer types [36]. This protective shield could also interfere with T‐cell trafficking within LP tumors, leading to the presentation of heterogeneous T‐cell response in LP tumors. Therefore, although the low and heterogeneous T‐cell response reflects the immune response to a heterogeneous mutational landscape, it is possible that other factors (such as stromal fibrosis) caused the cold and heterogeneous T‐cell response and led to ineffective immune surveillance, fostering genomic ITH in LP tumors. It is impossible to ascertain from these data whether the high level of genomic ITH led to profound heterogeneity in T‐cell response or vice versa. Nevertheless, the heterogeneous T‐cell response may lead to ineffective host anti‐tumor immune surveillance and a subsequent high recurrence rate. Additionally, emerging evidence has demonstrated that immune‐mediated cell death may play an important role in chemotherapy or radiotherapy‐induced cell death [46, 47]. The cold and heterogeneous immune microenvironment in LP could also contribute to poor response to chemotherapy and radiotherapy.

Currently, there are no effective targeted therapy agents in advanced LP [29]. High inter‐tumor and intratumor genomic ITH levels make it a challenge to find common clonal therapeutic targets. Recent unprecedented success with immune checkpoint blockade (ICB) has revolutionized the therapeutic landscape of many cancer types [48, 49]. Given the lack of effective treatment, there has been increasing enthusiasm for applying ICB to GC, including LP [50, 51, 52]. However, our findings of a high level of genomic ITH (i.e. the high proportion of branched mutations) consistent with the high ITH of gastric cancer [32, 53, 54, 55], together with low and heterogeneous T‐cell infiltration in LP tumors implies that ICB alone might be ineffective. Low complex genomic heterogeneity and less active T‐cell response are associated with inferior response to ICB [56]. Therefore, novel strategies are needed to optimize the efficacy of ICB in patients with LP. In addition to the standard chemotherapy, radiation and targeted therapy also exhibited improved efficacy of ICB [57, 58]. The fibrotic tumor stroma could be a potential therapeutic target to enhance response to ICB for LP. Preclinical studies have demonstrated that antifibrotic agents (e.g. pirfenidone, losartan, tranilast and hyaluronidase) could improve tumor perfusion, drug delivery and immune cell infiltration [59, 60, 61]. Therefore, antifibrotic therapy shows the potential to enhance T‐cell infiltration, promote intratumor T‐cell trafficking and subsequent T‐cell homogeneity, and thus improve the therapeutic efficacy of ICB [62, 63].

One major limitation of the current study was the limited sample size. Unfortunately, for rare tumors such as LP, surgical resection was only applicable to a small proportion of patients, thus, studying a large cohort of LP has turned out to be challenging. Despite this, the low T‐cell infiltration and high level of genomic and TCR ITH were observed across the four patients, which suggests these may be common biological features of LP tumors. For LP, collaborative efforts involving multiple centers are warranted to investigate its unique cancer biology to provide pivotal insight into the development of novel diagnostic and therapeutic strategies.

## 5 Conclusion

LP may have a great extent of genomic ITH and suppressed heterogeneous T‐cell infiltration, which may potentially be contributing factors to the high recurrence rate and poor therapeutic response. The suppressed immune microenvironment and a high degree of genomic/immune ITH suggest that mono‐immunotherapy may not be efficacious for LP.

## Conflict of interest

Dr. Jianjun Zhang reports grants from Merck, Johnson and Johnson; adversary/consulting/honoraria fees from Bristol Myers Squibb, AstraZeneca, Geneplus, Innovent, OrigMed and Roche outside the submitted work.

## Author contributions

JZ and ZC contributed to the conception or design of the work. JH, GZ, LJ, PL and JM interpreted the data and drafted the paper. GZ, LJ and JL carried out the bioinformatic analysis. JH, QP and JG acquired the data. YG, RC, XH, WCL, JM, XY, LC, AR, PAF and XX analyzed the data. All authors participated in the revisions and approved the final paper to be published.

### Peer review

The peer review history for this article is available at https://publons.com/publon/10.1002/1878‐0261.13381.

## Supporting information


**Fig. S1.** Comparison of TMB of the 40 LP tumors in this study vs. tumors from TCGA gastric cancer cohort.
**Fig. S2.** Trunk percentage of LP tumors.
**Fig. S3.** Mutational processes in LP tumors.
**Fig. S4.** Mutational signatures in LP tumors and stomach adenocarcinoma from TCGA.
**Fig. S5.** Mutational signatures in LP tumors.

**Table S1.** Clinicopathological characteristics of four patients with LP.

**Table S2.** Quality control of the whole exome sequencing data.

**Table S3.** Tumor purity for each sample.

## Data Availability

The raw DNA sequencing data were deposited in GSA‐human accessible ID: HRA000217 (URL: http://bigd.big.ac.cn/gsa‐human). All other analyzed data are supplied in supplementary materials and tables. The codes used and analyzed during the current study are available upon reasonable request.

## References

[1] Blackham AU , Swords DS , Levine EA , Fino NF , Squires MH , Poultsides G , et al. Is Linitis Plastica a contraindication for surgical resection: a multi‐institution study of the U.S. Gastric Cancer Collaborative. Ann Surg Oncol. 2016;23:1203–11. 26530447 10.1245/s10434-015-4947-8 PMC4980579

[2] Pedrazzani C , Marrelli D , Pacelli F , Di Cosmo M , Mura G , Bettarini F , et al. Gastric linitis plastica: which role for surgical resection? Gastric Cancer. 2012;15:56–60. 21717092 10.1007/s10120-011-0063-z

[3] Sah BK , Zhu ZG , Chen MM , Yan M , Yin HR , Zhen LY . Gastric cancer surgery and its hazards: post operative infection is the most important complication. Hepatogastroenterology. 2008;55:2259–63. 19260518

[4] Agnes A , Estrella JS , Badgwell B . The significance of a nineteenth century definition in the era of genomics: linitis plastica. World J Surg Oncol. 2017;15:123. 28679451 10.1186/s12957-017-1187-3 PMC5498981

[5] Chang JM , Lara KA , Gray RJ , Pockaj BA , Wasif N . Clinical outcomes after surgery for Linitis Plastica of the stomach: analysis of a population cancer registry. Am Surg. 2017;83:23–9. 28234115

[6] Swanton C . Intratumor heterogeneity: evolution through space and time. Cancer Res. 2012;72:4875–82. 23002210 10.1158/0008-5472.CAN-12-2217 PMC3712191

[7] Anaka M , Hudson C , Lo PH , Do H , Caballero OL , Davis ID , et al. Intratumoral genetic heterogeneity in metastatic melanoma is accompanied by variation in malignant behaviors. BMC Med Genomics. 2013;6:40. 24119551 10.1186/1755-8794-6-40 PMC3852494

[8] Lee HH , Kim SY , Jung ES , Yoo J , Kim TM . Mutation heterogeneity between primary gastric cancers and their matched lymph node metastases. Gastric Cancer. 2019;22:323–34. 30132154 10.1007/s10120-018-0870-6

[9] Sottoriva A , Spiteri I , Piccirillo SG , Touloumis A , Collins VP , Marioni JC , et al. Intratumor heterogeneity in human glioblastoma reflects cancer evolutionary dynamics. Proc Natl Acad Sci USA. 2013;110:4009–14. 23412337 10.1073/pnas.1219747110 PMC3593922

[10] Zhang J , Fujimoto J , Zhang J , Wedge DC , Song X , Zhang J , et al. Intratumor heterogeneity in localized lung adenocarcinomas delineated by multiregion sequencing. Science. 2014;346:256–9. 25301631 10.1126/science.1256930 PMC4354858

[11] Reuben A , Gittelman R , Gao J , Zhang J , Yusko EC , Wu CJ , et al. TCR repertoire intratumor heterogeneity in localized lung adenocarcinomas: an association with predicted neoantigen heterogeneity and postsurgical recurrence. Cancer Discov. 2017;7:1088–97. 28733428 10.1158/2159-8290.CD-17-0256 PMC5628137

[12] Richards S , Aziz N , Bale S , Bick D , Das S , Gastier‐Foster J , et al. Standards and guidelines for the interpretation of sequence variants: a joint consensus recommendation of the American College of Medical Genetics and Genomics and the Association for Molecular Pathology. Genet Med. 2015;17:405–24. 25741868 10.1038/gim.2015.30 PMC4544753

[13] Wu K , Zhang X , Li F , Xiao D , Hou Y , Zhu S , et al. Frequent alterations in cytoskeleton remodelling genes in primary and metastatic lung adenocarcinomas. Nat Commun. 2015;6:10131. 26647728 10.1038/ncomms10131 PMC4682110

[14] Shen R , Seshan VE . FACETS: allele‐specific copy number and clonal heterogeneity analysis tool for high‐throughput DNA sequencing. Nucleic Acids Res. 2016;44:e131. 27270079 10.1093/nar/gkw520 PMC5027494

[15] Rosenthal R , McGranahan N , Herrero J , Taylor BS , Swanton C . DeconstructSigs: delineating mutational processes in single tumors distinguishes DNA repair deficiencies and patterns of carcinoma evolution. Genome Biol. 2016;17:31. 26899170 10.1186/s13059-016-0893-4 PMC4762164

[16] Alexandrov LB , Kim J , Haradhvala NJ , Huang MN , Tian Ng AW , Wu Y , et al. The repertoire of mutational signatures in human cancer. Nature. 2020;578:94–101. 32025018 10.1038/s41586-020-1943-3 PMC7054213

[17] Murugaesu N , Wilson GA , Birkbak NJ , Watkins T , McGranahan N , Kumar S , et al. Tracking the genomic evolution of esophageal adenocarcinoma through neoadjuvant chemotherapy. Cancer Discov. 2015;5:821–31. 26003801 10.1158/2159-8290.CD-15-0412 PMC4529488

[18] De Sano L , Caravagna G , Ramazzotti D , Graudenzi A , Mauri G , Mishra B , et al. TRONCO: an R package for the inference of cancer progression models from heterogeneous genomic data. Bioinformatics. 2016;32:1911–3. 26861821 10.1093/bioinformatics/btw035 PMC6280783

[19] Mermel CH , Schumacher SE , Hill B , Meyerson ML , Beroukhim R , Getz G . GISTIC2.0 facilitates sensitive and confident localization of the targets of focal somatic copy‐number alteration in human cancers. Genome Biol. 2011;12:R41. 21527027 10.1186/gb-2011-12-4-r41 PMC3218867

[20] Roth A , Khattra J , Yap D , Wan A , Laks E , Biele J , et al. PyClone: statistical inference of clonal population structure in cancer. Nat Methods. 2014;11:396–8. 24633410 10.1038/nmeth.2883 PMC4864026

[21] Chen XX , Zhong Q , Liu Y , Yan SM , Chen ZH , Jin SZ , et al. Genomic comparison of esophageal squamous cell carcinoma and its precursor lesions by multi‐region whole‐exome sequencing. Nat Commun. 2017;8:524. 28900112 10.1038/s41467-017-00650-0 PMC5595870

[22] Bolotin DA , Poslavsky S , Mitrophanov I , Shugay M , Mamedov IZ , Putintseva EV , et al. MiXCR: software for comprehensive adaptive immunity profiling. Nat Methods. 2015;12:380–1. 25924071 10.1038/nmeth.3364

[23] Zhang X , Ai F , Li X , She X , Li N , Tang A , et al. Inflammation‐induced S100A8 activates Id3 and promotes colorectal tumorigenesis. Int J Cancer. 2015;137:2803–14. 26135667 10.1002/ijc.29671

[24] Nikbakht H , Panditharatna E , Mikael LG , Li R , Gayden T , Osmond M , et al. Spatial and temporal homogeneity of driver mutations in diffuse intrinsic pontine glioma. Nat Commun. 2016;7:11185. 27048880 10.1038/ncomms11185 PMC4823825

[25] Johnson BE , Mazor T , Hong C , Barnes M , Aihara K , McLean CY , et al. Mutational analysis reveals the origin and therapy‐driven evolution of recurrent glioma. Science. 2014;343:189–93. 24336570 10.1126/science.1239947 PMC3998672

[26] Eckert MA , Pan S , Hernandez KM , Loth RM , Andrade J , Volchenboum SL , et al. Genomics of ovarian cancer progression reveals diverse metastatic trajectories including intraepithelial metastasis to the fallopian tube. Cancer Discov. 2016;6:1342–51. 27856443 10.1158/2159-8290.CD-16-0607 PMC5164915

[27] Kim H , Zheng S , Amini SS , Virk SM , Mikkelsen T , Brat DJ , et al. Whole‐genome and multisector exome sequencing of primary and post‐treatment glioblastoma reveals patterns of tumor evolution. Genome Res. 2015;25:316–27. 25650244 10.1101/gr.180612.114 PMC4352879

[28] Abbosh C , Birkbak NJ , Wilson GA , Jamal‐Hanjani M , Constantin T , Salari R , et al. Phylogenetic ctDNA analysis depicts early‐stage lung cancer evolution. Nature. 2017;545:446–51. 28445469 10.1038/nature22364 PMC5812436

[29] Xue R , Li R , Guo H , Guo L , Su Z , Ni X , et al. Variable intra‐tumor genomic heterogeneity of multiple lesions in patients with hepatocellular carcinoma. Gastroenterology. 2016;150:998–1008. 26752112 10.1053/j.gastro.2015.12.033

[30] Jiang T , Shi T , Zhang H , Hu J , Song Y , Wei J , et al. Tumor neoantigens: from basic research to clinical applications. J Hematol Oncol. 2019;12:93. 31492199 10.1186/s13045-019-0787-5 PMC6731555

[31] Chen K , Yang D , Li X , Sun B , Song F , Cao W , et al. Mutational landscape of gastric adenocarcinoma in Chinese: implications for prognosis and therapy. Proc Natl Acad Sci USA. 2015;112:1107–12. 25583476 10.1073/pnas.1422640112 PMC4313862

[32] Pectasides E , Stachler MD , Derks S , Liu Y , Maron S , Islam M , et al. Genomic heterogeneity as a barrier to precision medicine in gastroesophageal adenocarcinoma. Cancer Discov. 2018;8:37–48. 28978556 10.1158/2159-8290.CD-17-0395 PMC5894850

[33] Sato N , Fukushima N , Chang R , Matsubayashi H , Goggins M . Differential and epigenetic gene expression profiling identifies frequent disruption of the RELN pathway in pancreatic cancers. Gastroenterology. 2006;130:548–65. 16472607 10.1053/j.gastro.2005.11.008

[34] Stein T , Cosimo E , Yu X , Smith PR , Simon R , Cottrell L , et al. Loss of reelin expression in breast cancer is epigenetically controlled and associated with poor prognosis. Am J Pathol. 2010;177:2323–33. 20847288 10.2353/ajpath.2010.100209 PMC2966791

[35] Dohi O , Takada H , Wakabayashi N , Yasui K , Sakakura C , Mitsufuji S , et al. Epigenetic silencing of RELN in gastric cancer. Int J Oncol. 2010;36:85–92. 19956836

[36] Castellano E , Molina‐Arcas M , Krygowska AA , East P , Warne P , Nicol A , et al. RAS signalling through PI3‐kinase controls cell migration via modulation of reelin expression. Nat Commun. 2016;7:11245. 27071537 10.1038/ncomms11245 PMC4833863

[37] Lee YS , Cho YS , Lee GK , Lee S , Kim YW , Jho S , et al. Genomic profile analysis of diffuse‐type gastric cancers. Genome Biol. 2014;15:R55. 24690483 10.1186/gb-2014-15-4-r55 PMC4056347

[38] Wang K , Yuen ST , Xu J , Lee SP , Yan HH , Shi ST , et al. Whole‐genome sequencing and comprehensive molecular profiling identify new driver mutations in gastric cancer. Nat Genet. 2014;46:573–82. 24816253 10.1038/ng.2983

[39] Cho SY , Park JW , Liu Y , Park YS , Kim JH , Yang H , et al. Sporadic early‐onset diffuse gastric cancers have high frequency of somatic CDH1 alterations, but low frequency of somatic RHOA mutations compared with late‐onset cancers. Gastroenterology. 2017;153:536–549.e26. 28522256 10.1053/j.gastro.2017.05.012 PMC6863080

[40] Wang R , Song S , Harada K , Ghazanfari Amlashi F , Badgwell B , Pizzi MP , et al. Multiplex profiling of peritoneal metastases from gastric adenocarcinoma identified novel targets and molecular subtypes that predict treatment response. Gut. 2020;69:18–31. 31171626 10.1136/gutjnl-2018-318070 PMC6943252

[41] Li X , Wu WK , Xing R , Wong SH , Liu Y , Fang X , et al. Distinct subtypes of gastric cancer defined by molecular characterization include novel mutational signatures with prognostic capability. Cancer Res. 2016;76:1724–32. 26857262 10.1158/0008-5472.CAN-15-2443

[42] Luo W , Fedda F , Lynch P , Tan D . CDH1 gene and Hereditary diffuse gastric cancer syndrome: molecular and histological alterations and implications for diagnosis and treatment. Front Pharmacol. 2018;9:1421. 30568591 10.3389/fphar.2018.01421 PMC6290068

[43] van der Post RS , Vogelaar IP , Carneiro F , Guilford P , Huntsman D , Hoogerbrugge N , et al. Hereditary diffuse gastric cancer: updated clinical guidelines with an emphasis on germline CDH1 mutation carriers. J Med Genet. 2015;52:361–74. 25979631 10.1136/jmedgenet-2015-103094 PMC4453626

[44] Zhang J , Qiu W , Liu H , Qian C , Liu D , Wang H , et al. Genomic alterations in gastric cancers discovered via whole‐exome sequencing. BMC Cancer. 2018;18:1270. 30567531 10.1186/s12885-018-5097-8 PMC6299976

[45] Morgant S , Artru P , Oudjit A , Lourenco N , Pasquer A , Walter T , et al. Computed tomography scan efficacy in staging gastric linitis plastica lesion: a retrospective multicentric French study. Cancer Manag Res. 2018;10:3825–31. 30288113 10.2147/CMAR.S163141 PMC6161744

[46] Golden EB , Apetoh L . Radiotherapy and immunogenic cell death. Semin Radiat Oncol. 2015;25:11–7. 25481261 10.1016/j.semradonc.2014.07.005

[47] Zhu Y , An X , Zhang X , Qiao Y , Zheng T , Li X . STING: a master regulator in the cancer‐immunity cycle. Mol Cancer. 2019;18:152. 31679519 10.1186/s12943-019-1087-y PMC6827255

[48] Anagnostou V , Smith KN , Forde PM , Niknafs N , Bhattacharya R , White J , et al. Evolution of neoantigen landscape during immune checkpoint blockade in non‐small cell lung cancer. Cancer Discov. 2017;7:264–76. 28031159 10.1158/2159-8290.CD-16-0828 PMC5733805

[49] Kohn CG , Zeichner SB , Chen Q , Montero AJ , Goldstein DA , Flowers CR . Cost‐effectiveness of immune checkpoint inhibition in BRAF wild‐type advanced melanoma. J Clin Oncol. 2017;35:1194–202. 28221865 10.1200/JCO.2016.69.6336 PMC5791832

[50] Cho J , Kang MS , Kim KM . Epstein‐Barr virus‐associated gastric carcinoma and specific features of the accompanying immune response. J Gastric Cancer. 2016;16:1–7. 27104020 10.5230/jgc.2016.16.1.1 PMC4834615

[51] Gros A , Robbins PF , Yao X , Li YF , Turcotte S , Tran E , et al. PD‐1 identifies the patient‐specific CD8(+) tumor‐reactive repertoire infiltrating human tumors. J Clin Invest. 2014;124:2246–59. 24667641 10.1172/JCI73639 PMC4001555

[52] Muro K , Chung HC , Shankaran V , Geva R , Catenacci D , Gupta S , et al. Pembrolizumab for patients with PD‐L1‐positive advanced gastric cancer (KEYNOTE‐012): a multicentre, open‐label, phase 1b trial. Lancet Oncol. 2016;17:717–26. 27157491 10.1016/S1470-2045(16)00175-3

[53] Lim CH , Cho YK , Kim SW , Choi MG , Rhee JK , Chung YJ , et al. The chronological sequence of somatic mutations in early gastric carcinogenesis inferred from multiregion sequencing of gastric adenomas. Oncotarget. 2016;7:39758–67. 27175599 10.18632/oncotarget.9250 PMC5129968

[54] Rocken C , Amallraja A , Halske C , Opasic L , Traulsen A , Behrens HM , et al. Multiscale heterogeneity in gastric adenocarcinoma evolution is an obstacle to precision medicine. Genome Med. 2021;13:177. 34749812 10.1186/s13073-021-00975-y PMC8576943

[55] von Loga K , Woolston A , Punta M , Barber LJ , Griffiths B , Semiannikova M , et al. Extreme intratumour heterogeneity and driver evolution in mismatch repair deficient gastro‐oesophageal cancer. Nat Commun. 2020;11:139. 31949146 10.1038/s41467-019-13915-7 PMC6965135

[56] McGranahan N , Furness AJ , Rosenthal R , Ramskov S , Lyngaa R , Saini SK , et al. Clonal neoantigens elicit T‐cell immunoreactivity and sensitivity to immune checkpoint blockade. Science. 2016;351:1463–9. 26940869 10.1126/science.aaf1490 PMC4984254

[57] Patel SA , Minn AJ . Combination cancer therapy with immune checkpoint blockade: mechanisms and strategies. Immunity. 2018;48:417–33. 29562193 10.1016/j.immuni.2018.03.007 PMC6948191

[58] Sharabi AB , Lim M , DeWeese TL , Drake CG . Radiation and checkpoint blockade immunotherapy: radiosensitisation and potential mechanisms of synergy. Lancet Oncol. 2015;16:e498–509. 26433823 10.1016/S1470-2045(15)00007-8

[59] Mpekris F , Papageorgis P , Polydorou C , Voutouri C , Kalli M , Pirentis AP , et al. Sonic‐hedgehog pathway inhibition normalizes desmoplastic tumor microenvironment to improve chemo‐ and nanotherapy. J Control Release. 2017;261:105–12. 28662901 10.1016/j.jconrel.2017.06.022 PMC5548264

[60] Polydorou C , Mpekris F , Papageorgis P , Voutouri C , Stylianopoulos T . Pirfenidone normalizes the tumor microenvironment to improve chemotherapy. Oncotarget. 2017;8:24506–17. 28445938 10.18632/oncotarget.15534 PMC5421866

[61] Stylianopoulos T . The solid mechanics of cancer and strategies for improved therapy. J Biomech Eng. 2017;139. 10.1115/1.4034991 PMC5248974 27760260

[62] Elahi‐Gedwillo KY , Carlson M , Zettervall J , Provenzano PP . Antifibrotic therapy disrupts stromal barriers and modulates the immune landscape in pancreatic ductal adenocarcinoma. Cancer Res. 2019;79:372–86. 30401713 10.1158/0008-5472.CAN-18-1334 PMC6335156

[63] Tacke F , Weiskirchen R . An update on the recent advances in antifibrotic therapy. Expert Rev Gastroenterol Hepatol. 2018;12:1143–52. 30261763 10.1080/17474124.2018.1530110

