# Supplementary material for: Analysis of genomic and immune intratumor heterogeneity in linitis plastica via multiregional exome and T‐cell receptor sequencing
Source: Mol Oncol. 2023 Mar 7;17(8):1531–44. doi: 10.1002/1878-0261.13381 (PMC10399711; doi:10.1002/1878-0261.13381)
Supplement: Supplementary file 1 — Fig. S1. Comparison of TMB of the 40 LP tumors in this study vs. tumors from TCGA gastric cancer cohort. Fig. S2. Trunk percentage of LP tumors. Fig. S3. Mutational processes in LP tumors. Fig. S4. Mutational signatures in LP tumors and stomach adenocarcinoma from TCGA. Fig. S5. Mutational signatures in LP tumors. [file MOL2-17-1531-s003.docx]

Supplementary figures for

Analysis of genomic and immune intratumor heterogeneity in linitis plastica via multiregional exome and T cell receptor sequencing

Jin Huang^1,2,3,4,5^, Guofeng Zhao^6,7^, Qiu Peng^8^, Xin Yi^6,7^, Liyan Ji^6,7^, Pansong Li^6,7^, Yanfang Guan^6,7,^, Jie Ge^3^, Ling Chen^3^, Runzhe Chen^9,10^, Xin Hu^9,10^, Won-Chul Lee^9^, Alexandre Reuben^9^, P. Andrew Futreal^10^,Xuefeng Xia^7^, Jian Ma^1,5,8,*^, Jianjun Zhang^9,10,*^, Zihua Chen^1,3,4,5,*^

Correspondence to: majian@csu.edu.cn

jzhang20@mdanderson.org

402899@csu.edu.cn


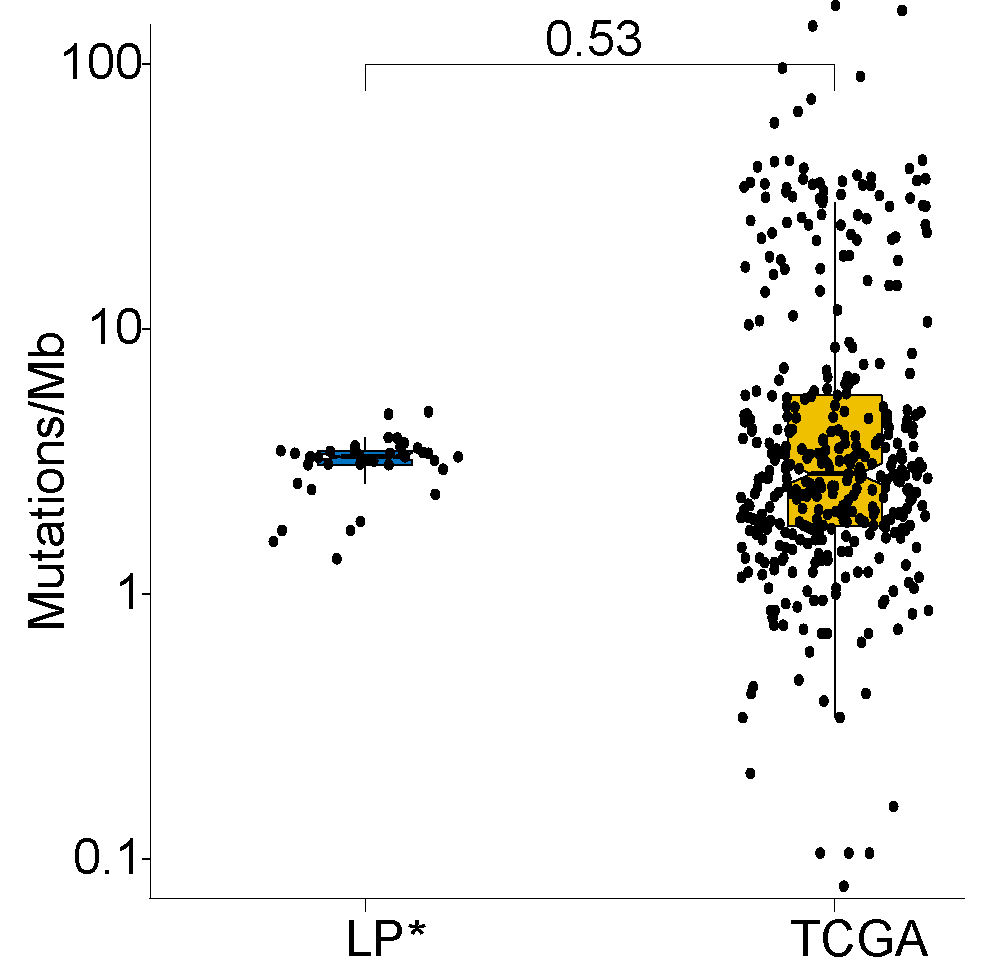


Figure. S1.

Comparison of TMB of the 40 LP tumors in this study versus tumors from TCGA gastric cancer cohort.


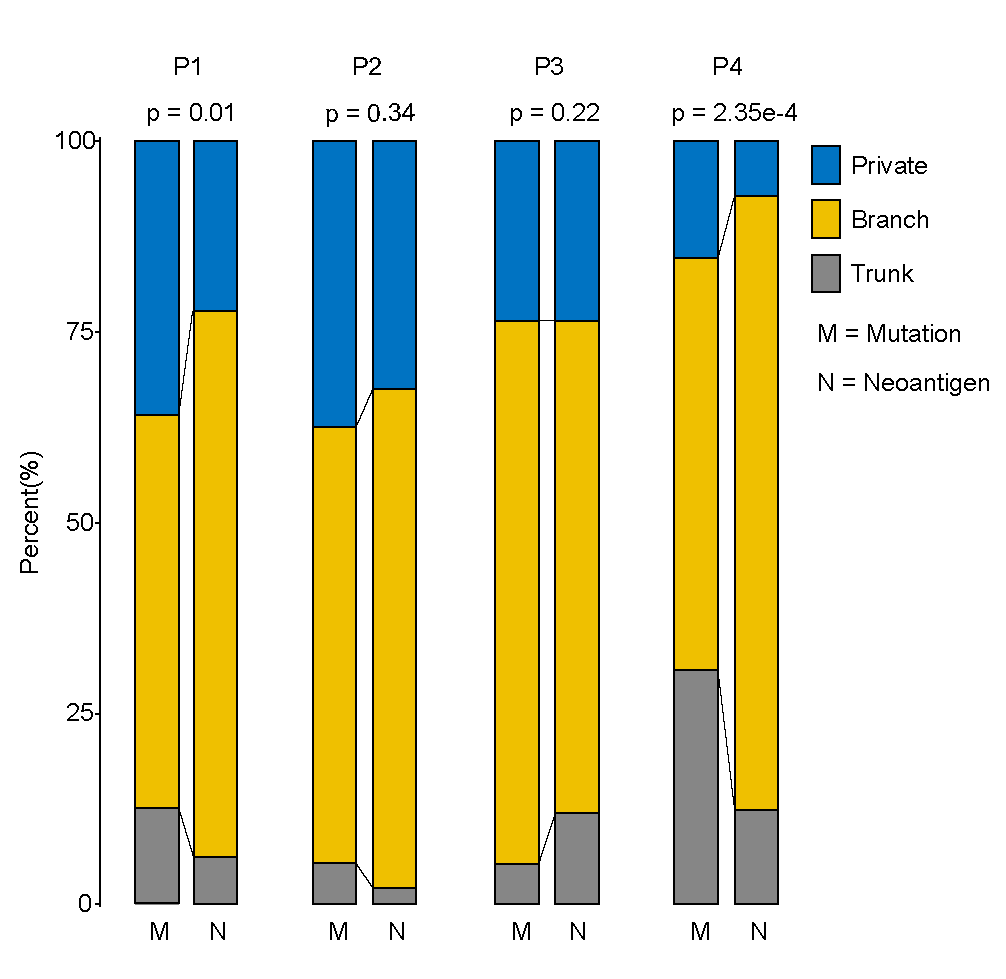


**Figure. S2.**

Trunk percent of LP tumors. The trunk percent of mutation(M) and neoantigen(N) of each LP tumor. Fisher’ test was used to calculate the difference between mutation trunk and neoantigen trunk of each LP tumor.


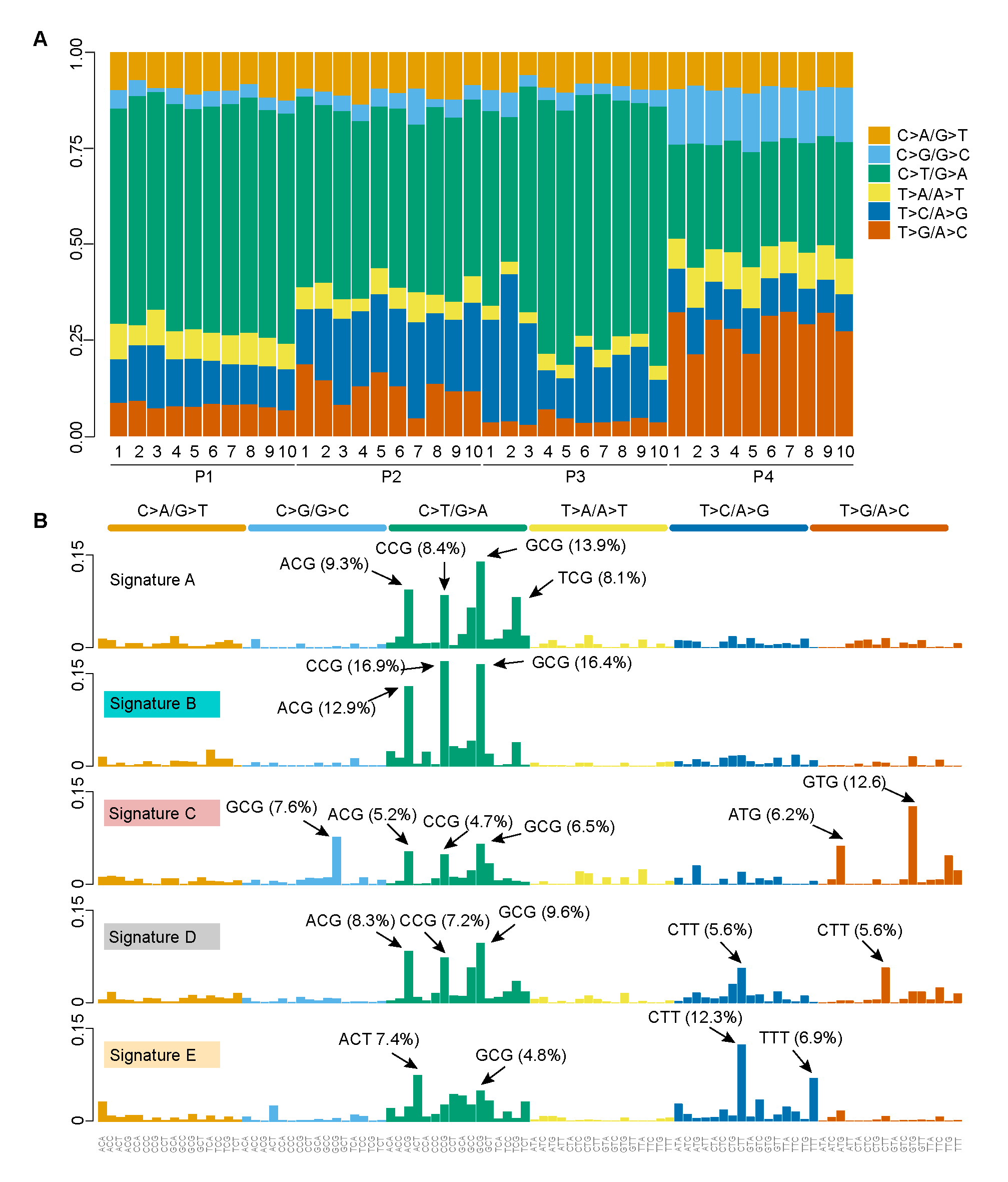
**Figure. S3.**

Mutational processes in LP tumors. (A) Mutation spectrum of transitions (Ti) and transversions (Tv) in 40 tumor regions from 4 LP tumors. (B) Mutation spectra of LP based on 96 base substitutes for the signatures obtained from NMF clustering.

**
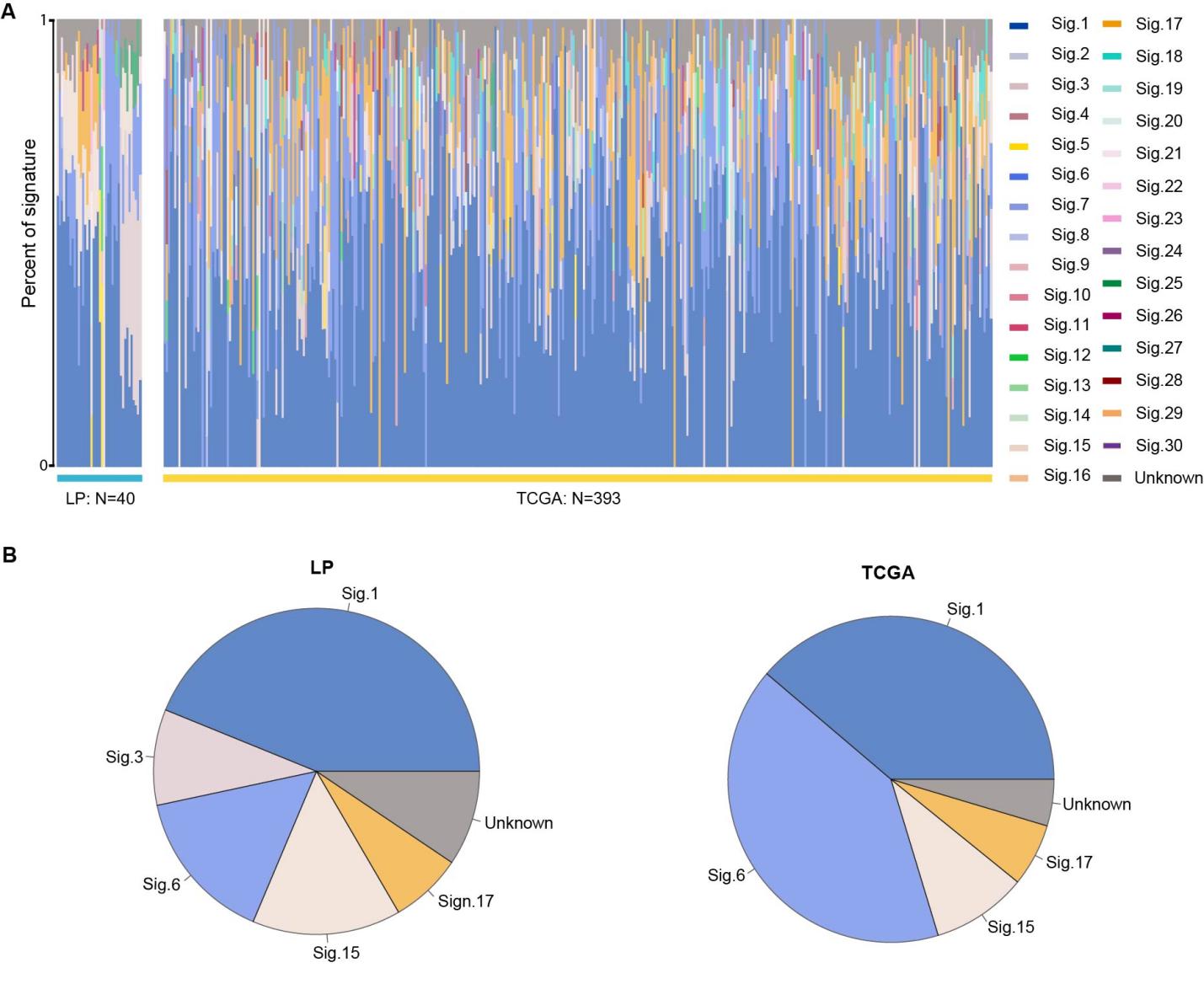
**

**Figure. S4.**

Mutational signatures in LP tumors and Stomach adenocarcinoma from TCGA. The contribution of different mutational signatures in each tumor (A) and group (LP vs TCGA) (B) using COSMICv2 signatures.

**
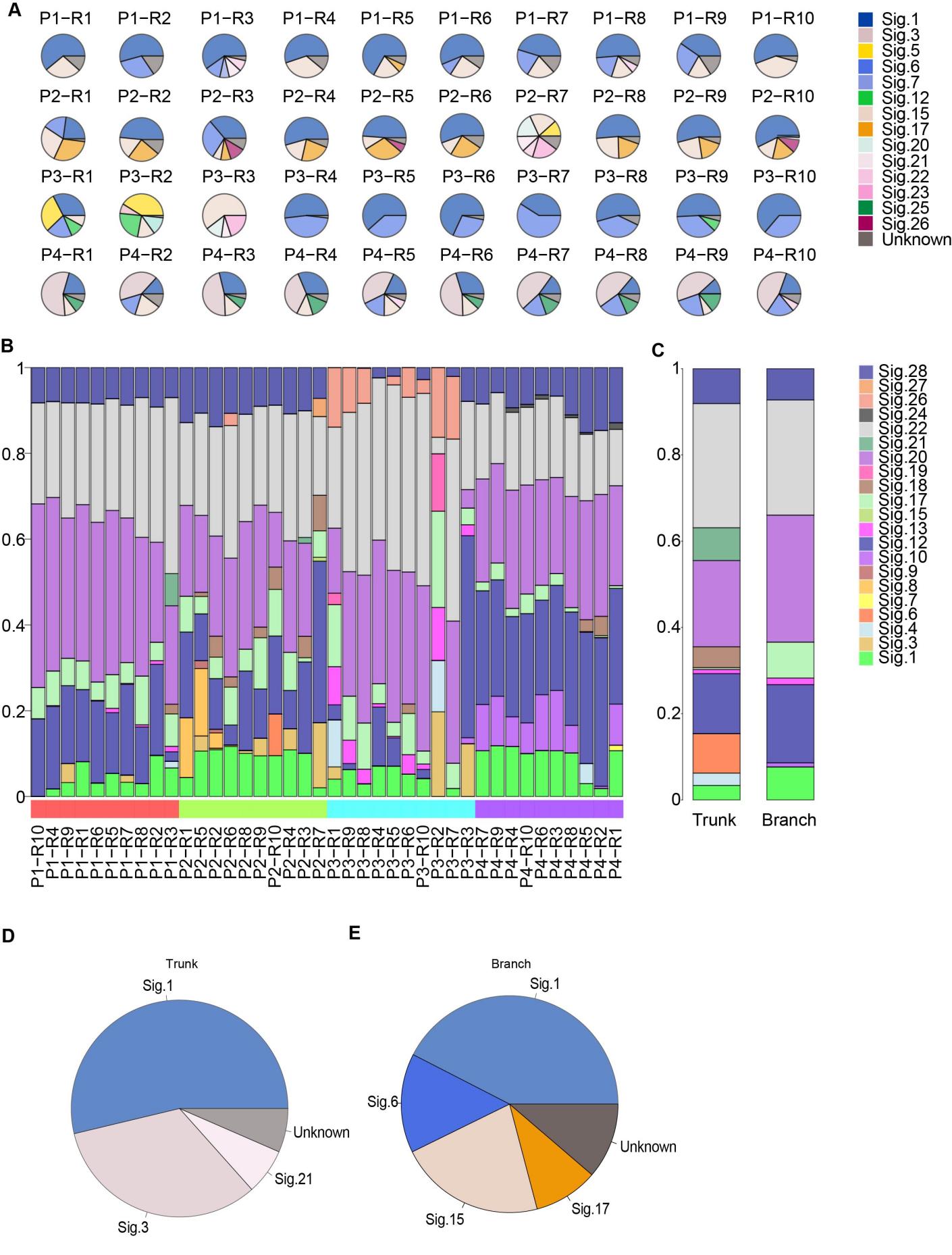
**

**Figure. S5.**

Mutational signatures in LP tumors. The mutational signatures for each sample using 30 COSMICv2 signatures using pie (A) and barplot (B). (C) The proportion of mutational signatures contributes to trunk and branch across all patients. (D-E) The contribution of different mutational signatures in each tumor and group (trunk/branch).
