# Supplementary material for: Analysis of genomic and immune intratumor heterogeneity in linitis plastica via multiregional exome and T‐cell receptor sequencing
Source: Mol Oncol. 2023 Mar 7;17(8):1531–44. doi: 10.1002/1878-0261.13381 (PMC10399711; doi:10.1002/1878-0261.13381)
Supplement: Supplementary file 3 — Table S2. Quality control of the whole exome sequencing data. [file MOL2-17-1531-s002.pdf]

Supplementaty Table S2. Quality control of the whole exome sequencing data.

Sample	Type	Average sequencing depth on target	Mapped reads on target	Total mapped reads	Total reads pass low quality check	Uniquely mapped reads on target	Total uniquely mapped reads	Fraction of target covered with >= 10x	Fraction of target covered with >= 50x
P1	normal	253.3	207954196	306554728	317061160	160986295	238893474	99.38%	97.47%
P1_R1	cancer	245.29	203043161	267587542	276587084	153560872	202845633	99.51%	97.10%
P1_R2	cancer	293.23	240455467	342444831	352497814	185458750	263426451	99.57%	98.06%
P1_R3	cancer	285.76	236342106	347700305	358707646	181919270	266884107	99.50%	98.04%
P1_R4	cancer	333.54	297252971	426200683	440483402	210984307	302169686	99.58%	98.46%
P1_R5	cancer	242.15	216083688	303928783	313010856	153085712	214330817	99.51%	97.00%
P1_R6	cancer	261.85	223668055	313676312	322923060	165501762	231122112	99.54%	97.38%
P1_R7	cancer	271.7	233988040	325257174	337605762	171272786	237543652	99.59%	97.74%
P1_R8	cancer	298.71	274906589	385123050	396937678	189382968	264071505	99.57%	98.05%
P1_R9	cancer	244.6	203753984	280229685	290091598	154055023	211222491	99.52%	97.25%
P1_R10	cancer	254.1	206006060	303802271	313560254	161317858	237010680	99.51%	97.51%
P2	normal	254.35	229284865	330044037	341498682	159841035	237832289	99.46%	97.31%
P2_R1	cancer	319.33	267457337	371722853	383519276	201246959	280108289	99.66%	98.51%
P2_R2	cancer	269.48	220175335	310789123	320600460	171101892	241513149	99.60%	97.98%
P2_R3	cancer	257.37	205881530	297578729	305017926	163574774	236097399	99.56%	97.72%
P2_R4	cancer	264.77	214252133	300624140	310388980	167190587	234427030	99.60%	97.64%
P2_R5	cancer	262.98	217664450	310409719	315165408	167203530	237518727	99.52%	97.43%
P2_R6	cancer	299.14	250312304	355617602	366636750	189676233	269509795	99.55%	98.18%
P2_R7	cancer	305.61	245656296	343061681	354922330	192519858	270118557	99.55%	98.18%
P2_R8	cancer	298.86	251590774	348114519	358439122	188192521	261365885	99.58%	98.07%
P2_R9	cancer	261.98	208434904	286389278	295507860	164605876	226705442	99.57%	97.42%
P2_R10	cancer	259.53	217323291	303075997	313354378	164200574	230067340	99.55%	97.73%
P3	normal	267.82	226485667	367795564	378430776	169385476	271521842	97.52%	85.19%
P3_R1	cancer	270.66	244765177	348809814	353674753	170092631	240722998	92.22%	78.24%
P3_R2	cancer	233.99	207656261	372974272	388904552	146277807	256097312	95.41%	76.61%
P3_R3	cancer	355.01	317261109	425684435	434348273	222446779	297635170	97.86%	91.71%
P3_R4	cancer	290.12	269816473	396374723	402862699	185115558	271053119	99.59%	98.17%
P3_R5	cancer	226.19	206051399	277637318	279436532	144497619	193417316	99.44%	96.54%
P3_R6	cancer	206.56	202461324	277034128	279657043	131290239	178536084	97.74%	87.23%
P3_R7	cancer	294.47	304257283	399052096	405069686	187220057	244709825	99.55%	98.10%
P3_R8	cancer	255.01	261497461	442419869	453013974	162387443	271840731	96.18%	83.68%
P3_R9	cancer	291.15	270933244	358066419	362723278	181032339	237559852	96.04%	88.25%
P3_R10	cancer	264.86	253400502	324277777	328630269	164799117	210260949	99.49%	96.07%
P4	normal	112.02	89254104	129747883	129891598	78162121	112995040	98.56%	82.28%
P4_R1	cancer	419.07	355538777	484222019	485543599	293708671	399505884	99.76%	99.07%
P4_R2	cancer	390.36	331196527	470818300	472013529	275033761	390202865	99.75%	98.93%
P4_R3	cancer	407.13	354468376	479466745	480590389	286222960	386987629	99.72%	98.84%
P4_R4	cancer	378.23	323102990	437713878	438967400	264417650	358068840	99.75%	98.91%
P4_R5	cancer	428.01	361780003	517108919	518305280	300917121	429389369	99.75%	99.02%
P4_R6	cancer	362.69	311820560	420098099	421121638	255432742	344255529	99.72%	98.69%
P4_R7	cancer	366.22	315215700	420971737	421972557	256340265	342318549	99.74%	98.86%
P4_R8	cancer	396.86	333451584	447664107	448966500	278312914	373250845	99.75%	99.01%
P4_R9	cancer	324	277715235	364807324	365778819	226588847	297583050	99.73%	98.65%
P4_R10	cancer	388.29	330633385	449956325	451121188	271772542	369906464	99.75%	98.94%

*note: **norrmal** represents the peripheral blood as control
